# Enhanced saccharification of rice straw by overexpression of rice exo-glucanase

**DOI:** 10.1186/1939-8433-5-14

**Published:** 2012-06-28

**Authors:** Mutsumi Nigorikawa, Aiko Watanabe, Kayoko Furukawa, Tomonori Sonoki, Yukihiro Ito

**Affiliations:** Graduate School of Agricultural Science, Tohoku University, 1-1 Tsutsumidori-Amamiyamachi, Aoba-ku, Sendai, 981-8555 Japan; Faculty of Agriculture and Life Science, Hirosaki University, Bunkyo-cho, Hirosaki, 036-8560 Japan

**Keywords:** Saccharification, Cell wall, Cellulase, Bioethanol, Rice

## Abstract

**Background:**

Efficient production of carbon-neutral biofuels is key to resolving global warming and exhaustion of fossil fuels. Cellulose, which is the most abundant biomass, is physically strong and biochemically stable, and these characteristics lead to difficulty of efficient saccharification of cellulosic compounds for production of fermentable glucose and other sugars.

**Results:**

We transformed rice with overexpressing constructs of rice genes encoding each of three classes of cellulases. The exo-glucanase overexpressing plants showed various abnormalities in leaf such as division of leaf blade, crack on leaf surface, excess lacunae in midrib structure and necrotic colour change. The overexpressing plants also showed sterility. Noticeably, these plants showed enhanced saccharification of stems after maturation. These results indicate that overexpression of the exo-glucanase gene brought about various developmental defects associated with modification of cell wall and enhanced saccharification in rice. On the other hand, endo-glucanase-overexpressing plants could not be obtained, and overexpression of β-glucosidase brought about no effect on plant growth and development.

**Conclusions:**

Our results indicate that genetic engineering of cellulosic biomass plants by overexpressing cellulase genes will be one of the approaches to confer enhanced saccharification ability for efficient production of cellulosic biofuels such as ethanol.

**Electronic supplementary material:**

The online version of this article (doi:10.1186/1939-8433-5-14) contains supplementary material, which is available to authorized users.

## Background

Efficient production of carbon-neutral biofuels is key to resolving global warming and exhaustion of fossil fuels. Cellulose is the most abundant biomass, and thus it is an indispensable and valuable material for the production of biofuels such as ethanol. However, plant cell wall, which contains cellulose, hemicellulose, pectins and lignins, is physically strong and biochemically stable, and these characteristics lead to difficulty of efficient saccharification of cellulosic compounds for production of fermentable glucose and other sugars (Hendriks and Zeeman[2009]).

To overcome this problem a number of studies have been carried out focusing on pre-treatment of cell wall to fractionate its components and remove lignins and on engineering microorganisms to enforce saccharification and fermentation abilities. Considering that food production has been greatly increased by breeding of crop plants, breeding of material plants is also indispensable for efficient production of biofuels. For this purpose a number of experiments have been carried out to accumulate cellulases in plants (Taylor et al.[2008]). These experiments utilized heterologous expression of microorganism-derived cellulases including thermophilic cellulases or cellulases targeted to specific organella such as chloroplasts to avoid deleterious effects on plant growth and development, and no plant-derived cellulase was used. In addition, whereas cellulases can be categorized into three classes of enzymes with different substrate specificities, endo-glucanase, exo-glucanase and β-glucosidase, only endo-glucanase was used in many cases. Thus, reliability of plant-derived cellulases and their homologous expression system as well as exo-glucanase and β-glucosidase remains to be elucidated.

In this study rice cellulases were overexpressed in rice. Our idea is that overexpression of cellulase in a living plant may lead to partial degradation and loosening of cell wall, and such cell wall can be an efficient substrate of cell wall-degrading enzymes produced by microorganisms by enhancing accessibility of the enzymes to the substrates during the saccharification process. To this end, we examined whether overexpression of cellulase can affect the structure of the cell wall and enhance its saccharification ability in rice. We also examined which class of cellulases is suitable for this purpose. We showed that overexpression of rice exo-glucanase resulted in various developmental defects associated with modified cell wall and enhanced saccharification in rice. We also showed that overexpression of endo-glucanase is deleterious to rice, and overexpression of β-glucosidase had no effect. Our results suggest that plant-derived exo-glucanase can be used for enhancing saccharification ability by overexpression.

## Results

### Generation of transgenic rice plants overexpressing *EXG1*

**Figure 1:**
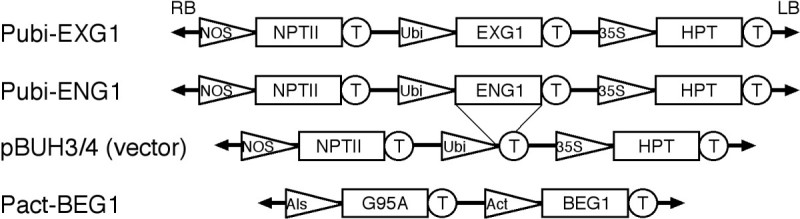
**Structure of the vectors used for rice transformation.** Triangles: a promoter for a nopaline synthase gene (NOS), maize ubiqutin gene (Ubi), cauliflower mosaic virus 35S RNA (35S), rice acetolactate synthase gene (Als) or rice actin gene (Act); boxes: a coding sequence of exo-glucanase (EXG1), endo-glucanase (ENG1), β-glucosidase (BEG1), neomycin phosphotransferase II (NPTII), hygromycin phosphotransferase (HPT) or rice mutated bispyribac sodium-resistant acetolactate synthase (G95A); circles: a terminator for a nopaline synthase gene (T). RB and LB indicate right border and left border of T-DNA, respectively.

To generate transgenic rice plants overexpressing an exo-glucanase gene, we made a binary vector construct in which rice full-length cDNA (AK108835) for exo-glucanase was placed between a maize ubiquitin promoter and a terminator of a nopaline synthase gene in a sense orientation (Figure 1). We referred to the gene for the AK108835 full-length cDNA as *EXG1*. The resultant construct was introduced into the rice genome by *Agrobacterium*-mediated transformation (Hiei et al.[1994]).

We first examined transformation efficiency, because overexpression of *EXG1* might have resulted in lethality to cells by degrading cellulose and inhibiting synthesis of the cell wall. We used about 200 calli for transformation with the *EXG1* construct (Pubi-EXG1) and obtained 15 transgenic plant lines (7.5%) in a cultivar Nipponbare and 12 lines (6%) in a cultivar Taichung 65 (Table 1). These efficiencies were comparable to those with an empty vector (6.5% in Nipponbare and 5% in Taichung 65) (Table 1). This suggests that overexpression of *EXG1* did not result in lethality to the cells.

**Table 1:** **Efficieny of transformation**

Construct	Vector	Protein	Cultivar	No of calli used	No of regenerated shoots	Effecicincy (%)
Pubi-EXG1	pBUH4	exo-glucanase	Nipponbare	200	15	7.5
Pubi-EXG1	pBUH4	exo-glucanase	Taichung 65	200	13	6.5
Pubi-ENG1	pBUH3	endo-glucanase	Taichung 65	600	0	0
Pact-BEG1	pSTARA380RALS (G95A)-GW	beta-glucosidase	Taichung 65	200	5	2.5
pBUH3	-	none	Nipponbare	200	13	6.5
pBUH3	-	none	Taichung 65	200	10	5

**Figure 2:**
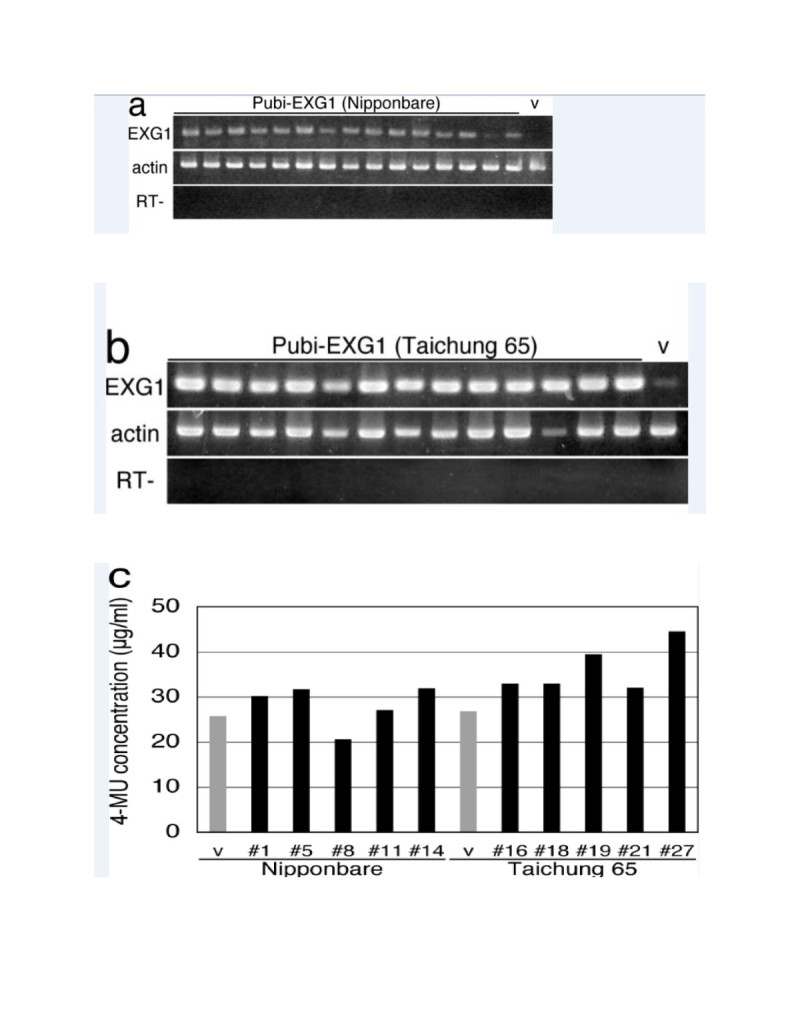
**Generation of the** ***EXG1*** **overexpressing plants. a** and **b** Expression of *EXG1* in the Pubi-EXG1 transgenic plants. RNAs isolated from leaves of the self-progenies of the Pubi-EXG1 primary transformants (Nipponbare in a and Taichung 65 in b) and the vector-transformed control plant were reverse-transcribed with the oligo(dT) primer and amplified by *EXG1* or actin specific primers. RT– indicates that reverse-transcriptase was omitted from the reaction mixture. **c** Cellulase activities of the Pubi-EXG plants. Protein extracts prepared from transgenic leaf blade of young seedlings were incubated with a fluorescent substrate 4-methylumbelliferyl β-D-cellobioside. v: a vector-transformed control plant.

We examined the expression of *EXG1* in the transgenic plants by RT-PCR using RNAs isolated from mature leaves. We observed high-level expression of *EXG1* in the Pubi-EXG1 transgenic plants, whereas the expression signal was barely detected in a control plant transformed with an empty vector (Figure 2a, b). We also examined the cellulase activities in protein extracts prepared from leaf blade of young seedlings of self-progenies of the primary transformants using the fluorescent substrate 4-methylumbelliferyl β-D-cellobioside. We observed higher fluorescence in the extracts prepared from the overexpressing plants than those prepared from the control plants (Figure 2c). These results indicate that the Pubi-EXG1 plants retained high cellulase activities.

### Morphological effects of overexpression of *EXG1*

**Figure 3:**
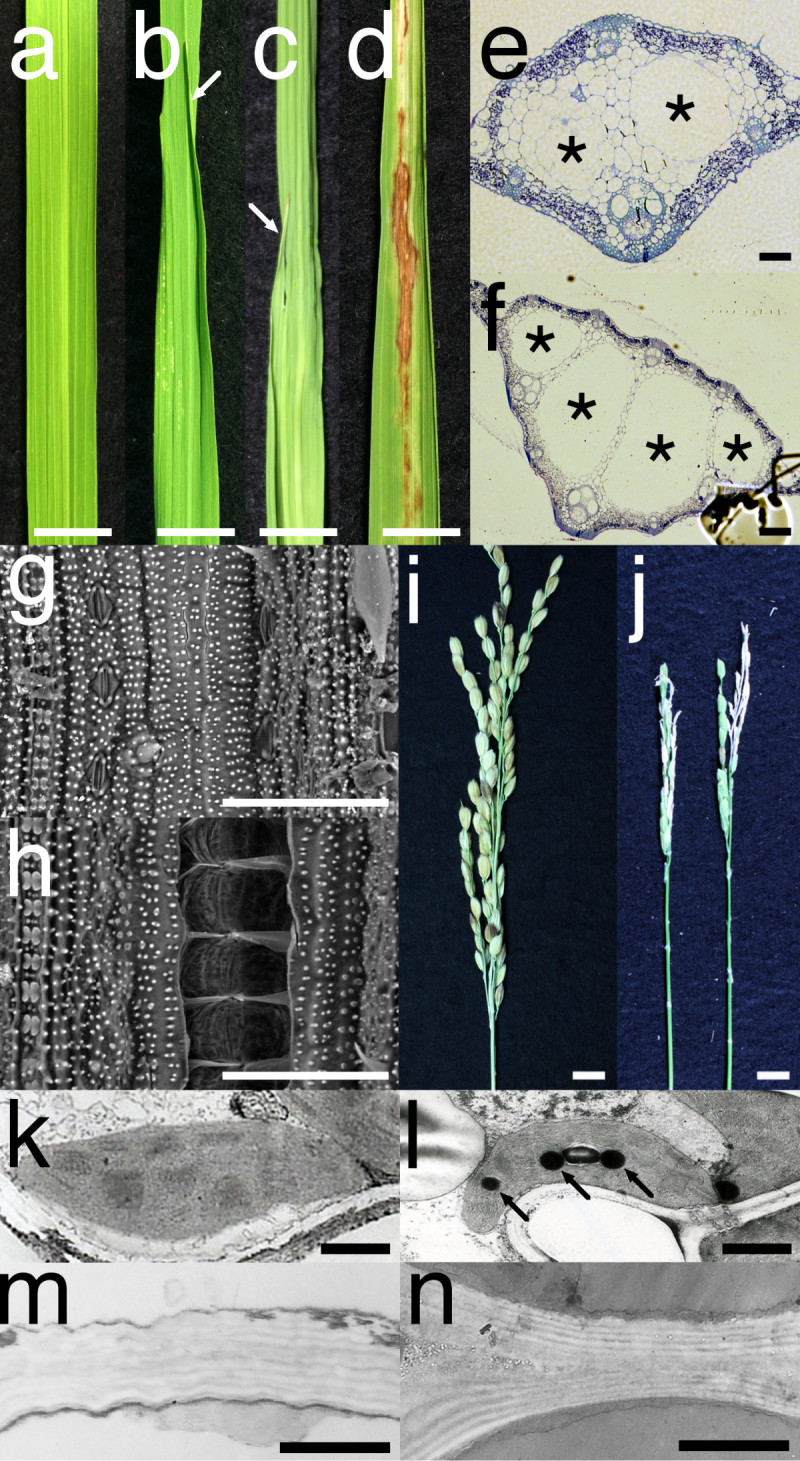
**Phenotypes of the** ***EXG1*** **-overexpressing plants. a** Wild type leaf blade. **b**, **c** Division of leaf blade observed in the Pubi-EXG1 #4 (b) and #13 (c) plants (Nipponbare). Arrows indicate divided small leaf blade. **d** Necrotic colour change in leaf blade of the Pubi-EXG1 #10 plant (Nipponbare). **e**, **f** A transverse section of the leaf blade with two lacunae in the midvein of the control plant (e) or the leaf blade with four lacunae in the midvein of the Pubi-EXG1 #18 plant (Taichung 65) (f). Asterisks indicate lacunae. **g**, **h** Simplified SEM observation of the surface of the leaf blade of the control plant (g) or the Pubi-EXG1 #18 plant (h). **i**, **j** Panicle of the control plant (i) and the Pubi-EXG1 #18 plant (j). **k**, **l** TEM observation of chloroplast of the leaf blade of the control plant (k) or the Pubi-EXG1 #18 plant (l). Arrows indicate plastogranules. **m**, **n** Cell wall of the control plant (m) and the Pubi-EXG1 #18 plant (n). Bars = 1 cm in (a) to (d), (i) and (j), 100 μm in (e) to (h), and 1 μm in (k) to (n).

We examined the effects of overexpression of *EXG1* on rice development. Although we did not observe any morphological and physiological abnormalities during transformation and shoot regeneration processes, we did observe various developmental defects after the transfer of regenerated plants to soil. Out of the 28 *EXG1*-overexpressing plants examined, three had leaves in which a part of the leaf blade was separated into two to form a larger leaf blade and a smaller leaf blade (Figure 3a to c). Leaf blade separation started from the middle of the leaf blade along the proximo-distal axis, and a smaller division leaf lacked a midvein and was twisted gradually so it was perpendicular to the other larger division leaf with the midvein (Figure 3a to c).

We further examined the morphology of the Pubi-EXG1 plants by microscope observation. In paraffin sections of the leaf blade, four lacunae were observed in the midvein of the three Pubi-EXG1 plants out of three examined, whereas the control plant showed two lacunae (Figure 3e,f). In simplified scanning electron microscope observation, small cracks on the adaxial surface of leaf blade were often observed in two Pubi-EXG1 plants examined, but very few were observed in the control plant (Figure 3g,h). These cracks were probably caused by vacuum during SEM observation. These phenotypes were assumed to be due to the weakened cell wall caused by the enhanced cellulase activity in the *EXG1*-overexpressing plants.

We observed necrotic colour change of the leaf blade in 24 plants out of 28 (Figure 3a,d). Although we generally observed colour change at the tip of the leaf blade when the leaf became senescent in the control plant, the colour change in the *EXG1*-overexpressing plants was observed even at the middle of the leaf blade before appearance of the colour change at the tip (Figure 3d). Transmission electron microscope observation of the Pubi-EXG1 plant (#18) showed that chloroplasts in its leaves had many plastogranules, which generally formed along with senescence (Maeda and Miyake[1993]). The vector-transformed control plant at the same stage had few or no plastogranules in the chloroplasts (Figure 3k,l). No difference of structure or thickness of cell wall was observed between the Pubi-EXG1 plant and the control plant (Figure 3m,n). These results suggest that overexpression of *EXG1* enhances the senescence of the leaf.

In addition to these phenotypes observed in the vegetative phase, the Pubi-EXG1 plants had small panicle and showed sterility. Out of the 28 Pubi-EXG1 plants, 12 were completely sterile and 14 were partially sterile (Figure 3i,j). The partially sterile plants produced less than 40 seeds per plant.

### Enhanced saccharification of the transgenic rice plants

**Figure 4:**
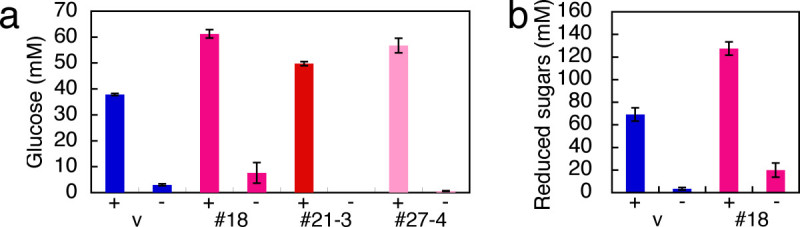
**Saccharification of the** ***EXG1*** **-overexpressing plants. a** Glucose. **b** Reduced sugars. + and – indicate whether cellulases were added to or omitted from the reaction mixture. #18: the primary transformant of Pubi-EXG1 (Taichung 65), #21-3 and #27-4: the self-progenies of the primary transformants of Pubi-EXG1 (Taichung 65), v: the vector transformed control plant.

We examined the saccharification efficiency of stems of the Pubi-EXG1 plants. The result showed that the Pubi-EXG1 plants yielded more glucose and reduced sugars than the control plant (Figure 4). This demonstrates that overexpression of *EXG1* resulted in enhanced saccharification to rice stem.

### A deleterious effect of overexpression of *ENG1*

We generated a construct overexpressing an endo-glucanase gene *ENG1* under the control of the ubiquitin promoter (Pubi-ENG1) (Figure 1), and introduced it into the rice genome. Although we used a total of 600 calli in three independent transformation experiments, no regeneration of shoots was observed even by a prolonged culture on a regeneration medium (Table 1). This suggests that overexpression of *ENG1* is deleterious to rice cells.

### Generation and morphology of transgenic rice plants overexpressing *BEG1*

We generated a construct overexpressing β-glucosidase gene *BEG1* under the control of the actin promoter (Pact-BEG1) (Figure 1) and introduced it into the rice genome. From 200 calli used for the transformation, we obtained 5 transformants (Table 1). RT-PCR analysis confirmed overexpression of *BEG1* in the Pact-BEG1 transgenic plants (Additional file1: Figure S1). We also examined the morphologies of the Pact-BEG1 transgenic plants. They grew normally and set seeds. No difference from the control plant was observed. Thus, the overexpression of *BEG1* barely affected transformation frequency or plant growth.

## Discussion

In this study we successfully generated transgenic rice plants with enhanced saccharification ability by overexpressing exo-glucanase *EXG1* derived from rice itself. Several previous studies have shown accumulation of thermostable endo-glucanase derived from thermophilic microorganisms and saccharification after heat treatment (Oraby et al.[2007]; Ransom et al.[2007]). Accumulation of endo-glucanase in chloroplasts was also reported (Mahadevan et al.[2011]). These studies aimed to avoid unsuitable effects of overexpression of cell wall degrading enzymes during plant growth and to saccharify cell wall components after harvesting the material plants by heating or destruction of chloroplasts. In contrast, we aimed to enhance the saccharification ability of a living material plant before harvesting and thereby omit any additional treatment necessary for activation of accumulated cellulases. In addition, we used a rice exo-glucanase gene for overexpression. The use of a crop-derived gene and its introduction into a donor species should help gain wider public acceptance of genetically modified organisms. In addition, problems associated with a heterologous expression system, such as splicing of cryptic introns and inappropriate codon usage, could also be avoided. We showed the reliability of overexpression of exo-glucanase for enhancing saccharification ability. On the other hand, constitutive overexpression of the rice exo-glucanase resulted in various developmental defects, which were necessary to overcome and are discussed below.

We examined which class of rice cellulases is most suitable for acquiring enhanced saccharification ability to rice plant by overexpression. We assumed two antagonistic possibilities of the effects of the overexpression of each class of cellulases. One is that overexpression of cellulase results in the degradation of cell wall and leads to lethality of the cell. The overexpression of *ENG1* is assumed to be the situation in this case. The other possibility is that overexpression of a single degrading enzyme of the cell wall components do not affect the structure of the cell wall, since the rice cell wall consists of various components and is very complex, and thus cellulase cannot successfully and sufficiently access its substrate cellulose which is covered with other components such as lignins. The analyses of the *EXG1*-overexpressing plants, however, showed that neither possibility is the case for an exo-glucanase gene. We observed no reduced frequency of transformation of callus with the *EXG1*-overexpressing construct, which should have been seen if the *EXG1* overexpression had brought about lethality to a cell. Instead, we obtained the transgenic plants which overexpressed introduced *EXG1* and retained high cellulase activity. We observed various developmental defects in the *EXG1*-overexpressing plants, and these defects seemed to be associated with cell wall defects. For example, leaf divisions may have been caused by an insufficient cell wall formation, which led to the separation of the tissues of the leaf blade during its development. The cracks on the leaf surface also seemed to be caused by a weakened cell wall. These results indicate that expression of exo-glucanase is sufficient to modify, but not significantly degrade, the structure of the cell wall. Noticeably, we showed that the *EXG1*-overexpressing plants had enhanced saccharification ability. Thus, breeding of material plants suitable for biofuel production can be achieved by genetic engineering by use of plant-derived exo-glucanase.

In contrast to the effects of overexpression of exo-glucanase or endo-glucanase, the overexpression of β-glucosidase had no effect on plant growth. Since β-glucosidase is an enzyme which hydrolyses disaccharide cellobiose, its overexpression did not affect directly the structure of the cell wall, and thus no abnormal morphology was observed. This indicates that overexpression of β-glucosidase alone is not sufficient to enhance saccharification of cellulosic biomass, but it is still possible that a combination of overexpression of β-glucosidase with exo-glucanase may further enhance saccharification efficiency. This possibility remains to be elucidated.

We used rice as a model plant. Since this enhancement of saccharification ability by genetic engineering depends only on a genetic transformation technique, this approach is not limited to rice, but it can be applied to various other crop plants such as maize, wheat and barley, and also to biomass plants such as switchgrass in which a genetic transformation system is available.

The use of rice straw for a biofuel material is an alternative way to avoid competition with feeding humans or other uses because a large amount of rice straw is produced every year around the world, and most rice straw is not used extensively (Kim and Dale[2004]). We used the constitutive maize ubiqutin promoter for the expression of *EXG1*, and this resulted in various developmental defects unsuitable for normal plant growth and seed production. These unfavourable phenotypes somehow need to be removed in order to utilize rice plants for both food production and biofuel production. The use of an inducible promoter is one of the ways to resolve this problem (Kasuga et al.[1999]). Additional studies are necessary to develop a better strategy to engineer food-biofuel dual-use plants and to generate a plant more suitable for biofuel production as well as food production.

## Conclusions

Our study showed the reliability of overexpression of rice-derived exo-glucanase for enhancing saccharification ability of rice straw and indicated that genetic engineering of cellulosic biomass plants by overexpressing cellulase genes will be one of the approaches to confer enhanced saccharification ability for efficient production of cellulosic biofuels such as ethanol. However, overexpression of exo-glucanase also brought about unfavoured phenotypes such as abnormal morphologies and sterility, which need to be resolved by future studies to generate food-biofuel dual-use plants.

## Methods

### Plasmid construction and rice transformation

To generate pBUH3 and pBUH4 binary vector plasmids, *Bam* HI and *Hin* dIII sites were removed from a binary vector plasmid pBCH1 (Ito et al.[2001]) by cutting these sites with corresponding restriction enzymes and re-ligation after filling-in. Then, the maize ubiqutin promoter was amplified by PCR, and the cauliflower mosaic virus 35S promoter of pBCH1 was replaced. The resulting plasmids pBUH3 and pBUH4 contained unique restriction sites of *Sac* I, *Spe* I, *Bam* HI, *Sma* I, *Hin* dIII and *Kpn* I with this order in pBUH3 and with the opposite order in pBUH4 between the maize ubiquitin promoter and a terminator of a nopaline synthase gene. These vectors harboured a hygromycin resistance gene as a selection marker gene.

A full-length cDNA (AK108835) for *EXG1* of rice (*Oryza sativa*), which is annotated to encode exo-glucanase in a rice full-length cDNA database (KOME database:http://cdna01.dna.affrc.go.jp/cDNA/), was inserted into the multi-cloning site of pBUH4. The resultant plasmid contained cDNA between the maize ubiquitin promoter and the terminator of a nopaline synthase gene in a sense orientation (Figure 1). A full-length cDNA (AK102748) for *ENG1* of rice, which is annotated to encode endo-glucanase, was inserted into the multi-cloning site of pBUH3. The resultant plasmid contained cDNA between the maize ubiquitin promoter and the terminator of a nopaline synthase gene in a sense orientation (Figure 1).

The overexpressing construct of β-glucosidase was generated using a Gateway system (Invitrogen). An entire coding sequence of a full-length cDNA (AK070962) for *BEG1* of rice, which is annotated to encode β-glucosidase, was amplified with *BEG1*-specific primers (BEG1-F1cut: 5’-CACCATGGCAGCTGCAGGGGAA-3’ and BEG1-R1: 5’-GGTGAGCCGTGCTCAAGGAG-3’), and cloned into pENTR (Invitrogen). Then, the cDNA was cloned into pSTARA380RALS(G95A)-GW (provided from Kumiai Chemical Industry) by LR clonase according to the manufacturer’s protocol. The resultant plasmid contained cDNA between the rice actin promoter and the terminator of a nopaline synthase gene in a sense orientation (Figure 1). This vector harboured a herbicide bispyribac sodium (BS) resistance gene as a selection marker gene (Okuzaki et al.[2007]).

*Agrobacterium tumefaciens* EHA101 was transformed with the binary vectors. *Agrobacterium*-mediated transformation of rice was carried out as described by Hiei et al. ([1994]). Rice (*Oryza sativa*) japonica cultivars, Nipponbare and Taichung 65, were used. Transgenic rice plants were grown in a green house at 27°C under natural sunlight.

### Expression analysis

RNA isolation, poly(A)^+^RNA purification and RT-PCR were carried out as described previously (Ito et al.[2001]). *EXG1* specific primers (EXG1-F1: 5’- GAGTTCGCCAACTCTTCCGA -3’ and EXG1-R1: 5’- AAGAGCGGGCTTAGCGTTCT -3’) and *BEG1* specific primers (BEG1-F2: 5’-CTGTGTTGCGGGATATGCAT-3’, and BEG1-R1) were used. Actin was used as an internal control and amplified with the primers RAc-1 (5’-AACTGGGATGATATGGAGAA-3’) and RAc-2 (5’-CCTCCAATCCAGACACTGTA-3’).

### Cellulase activity

Seeds obtained by self-fertilization of primary transformants were germinated and grown on an MS medium supplemented with 3% sucrose at 30°C under continuous light. Leaf blades of the 2 to 3-week-old seedlings were homogenized in a 2 ml buffer (50 mM phosphate buffer (pH 7.0), 10 mM EDTA (pH 8.0), 0.1% Triton X-100, 0.1% sarcosyl, 10 mM mercaptoethanol, 20% methanol) to prepare total protein extracts. The cellulase reaction mixture contained 0.5 μg of the total protein in the same buffer supplemented with a fluorescent substrate 4-methylumbelliferyl β-D-cellobioside, and incubated at 37°C for 6 h. The fluorescent signal was detected with a fluorometer.

### Saccharification

Rice culms at a maturation stage were dried at 105°C for 1 to 2 h, and were ground into powder. The powder was fractionated with mesh to collect particles at sizes less than 77 μm in diameter. The samples (30 mg each) were incubated at 50°C for 24 h in a 2 ml reaction mixture containing 100 mM sodium citrate (pH 4.8), 0.06 FPU of Celluclast 1.5 L (Sigma-Aldrich) and 0.24 units of Novozyme 188 (Sigma-Aldrich). Reduced sugars were measured with the DNS method (Sumner[1921]), and glucose was measured using a Biosensor-BF5 (Oji Scientific Instruments).

### Microscope observation

Leaf blade of mature plants grown in a green house at 27°C under natural sunlight was used for microscope observations. The surface of the leaf blade was observed using a simplified scanning electron microscope, Miniscope TM1000 (Hitachi, Japan). Standard paraffin sections of the leaf blade were prepared and observed with a conventional optical microscope. Transmission electron microscope observation was entrusted to Ultrastructure Research Laboratories, Inc., Japan. Briefly, leaf blade samples were fixed with glutaraldehyde solution and then osmium oxide. The fixed samples were dehydrated with gradual ethanol series, imbibed in propylene oxide and embedded in Spurr resin. After sectioning, the samples were stained with uranyl acetate and lead and observed with a JEOL JEM 100 S electron microscope at 80 kV (Miyoshi et al.[2003]).

## Electronic supplementary material

Additional file 1:**Figure S1.** Expression of *BEG1* in the *BEG1*-transgenic plants. RNAs isolated from leaves of the primary transformants of Pact-BEG1 (Taichung 65) and the vector-transformed control plant (v) were reverse-transcribed with the oligo(dT) primer and amplified by *BEG1* or actin specific primers. RT– indicates that reverse-transcriptase was omitted from the reaction mixture. (TIFF 1430 kb) (TIFF 1 MB)

Below are the links to the authors’ original submitted files for images.

Authors’ original file for figure 1

Authors’ original file for figure 2

Authors’ original file for figure 3

Authors’ original file for figure 4

Authors’ original file for figure 5
