# Supplementary figures and images for: Enhanced saccharification of rice straw by overexpression of rice exo-glucanase
Source: Rice (N Y). 2012 Jun 28;5:14. doi: 10.1186/1939-8433-5-14 (PMC4883724; doi:10.1186/1939-8433-5-14)

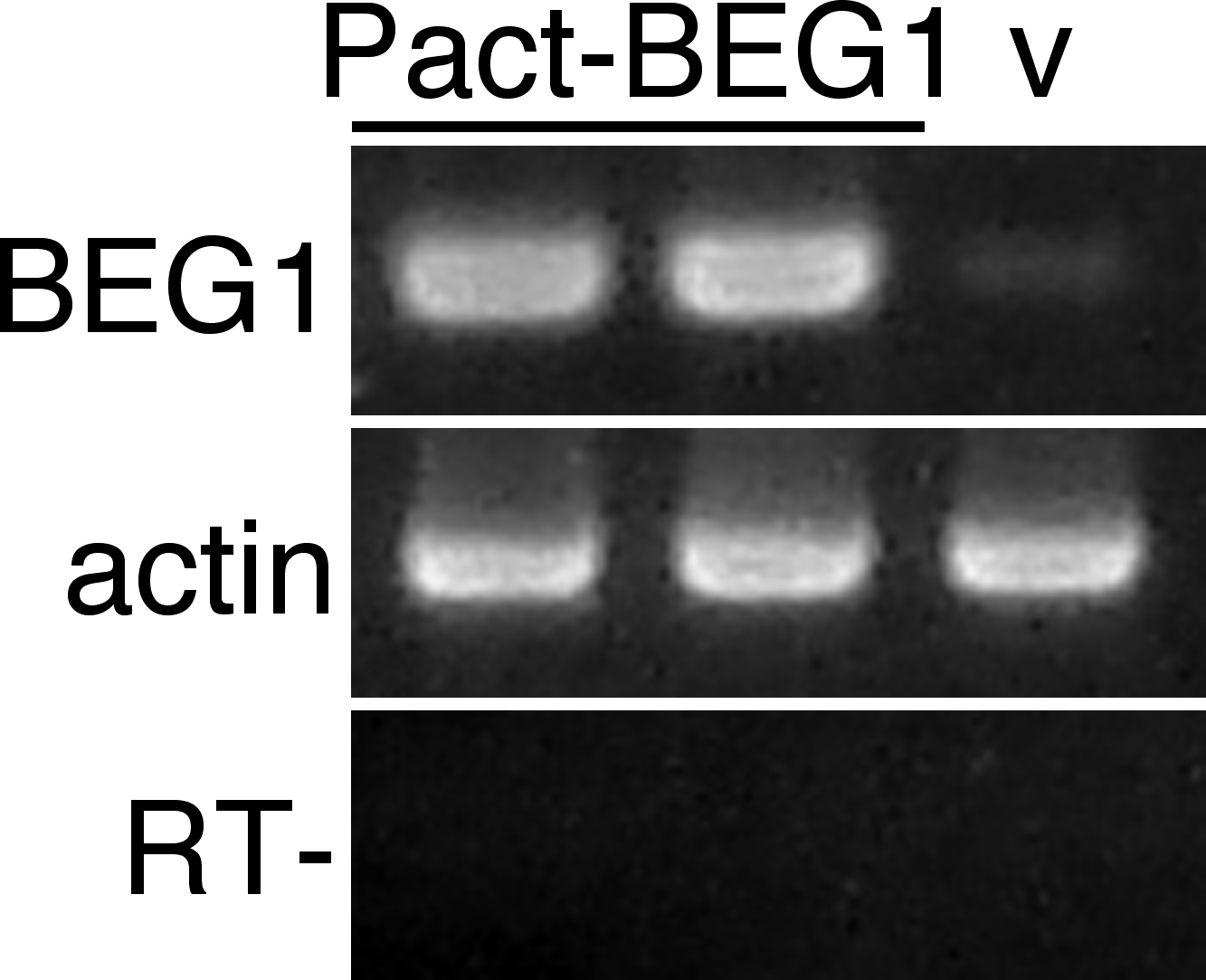

Supplement: Supplementary file 1 — Additional file 1:Figure S1. Expression of BEG1 in the BEG1-transgenic plants. RNAs isolated from leaves of the primary transformants of Pact-BEG1 (Taichung 65) and the vector-transformed control plant (v) were reverse-transcribed with the oligo(dT) primer and amplified by BEG1 or actin specific primers. RT– indicates that reverse-transcriptase was omitted from the reaction mixture. (TIFF 1430 kb) (TIFF 1 MB) [file 12284_2012_25_MOESM1_ESM.tiff]

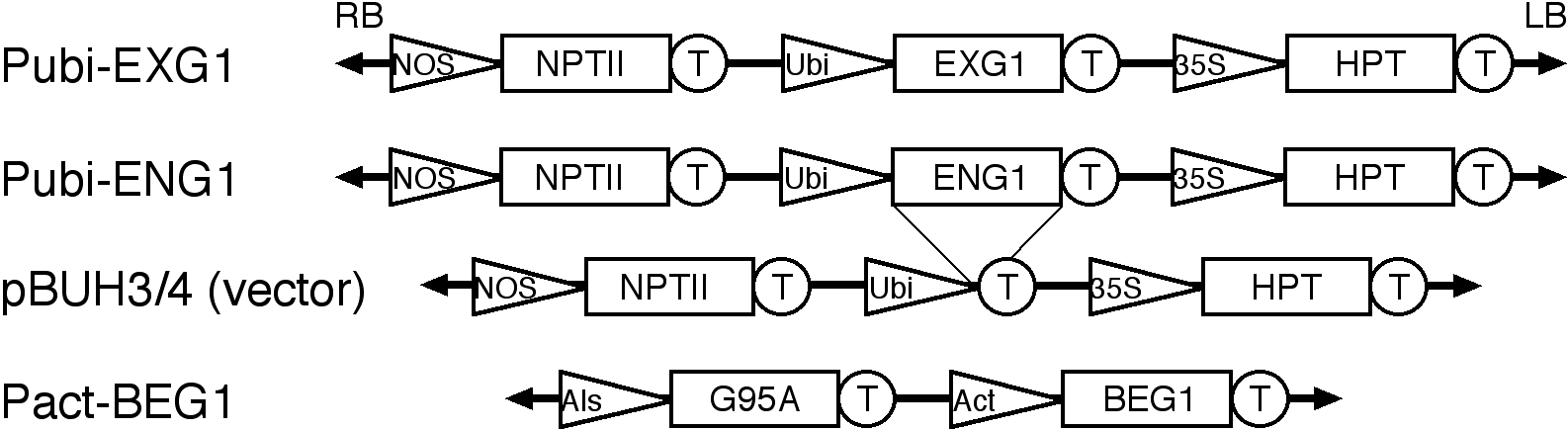

Supplement: Supplementary file 2 — Authors’ original file for figure 1 [file 12284_2012_25_MOESM2_ESM.tiff]

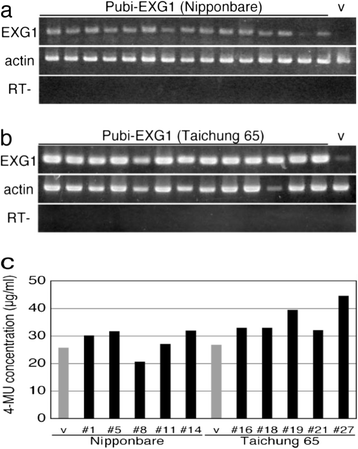

Supplement: Supplementary file 3 — Authors’ original file for figure 2 [file 12284_2012_25_MOESM3_ESM.gif]

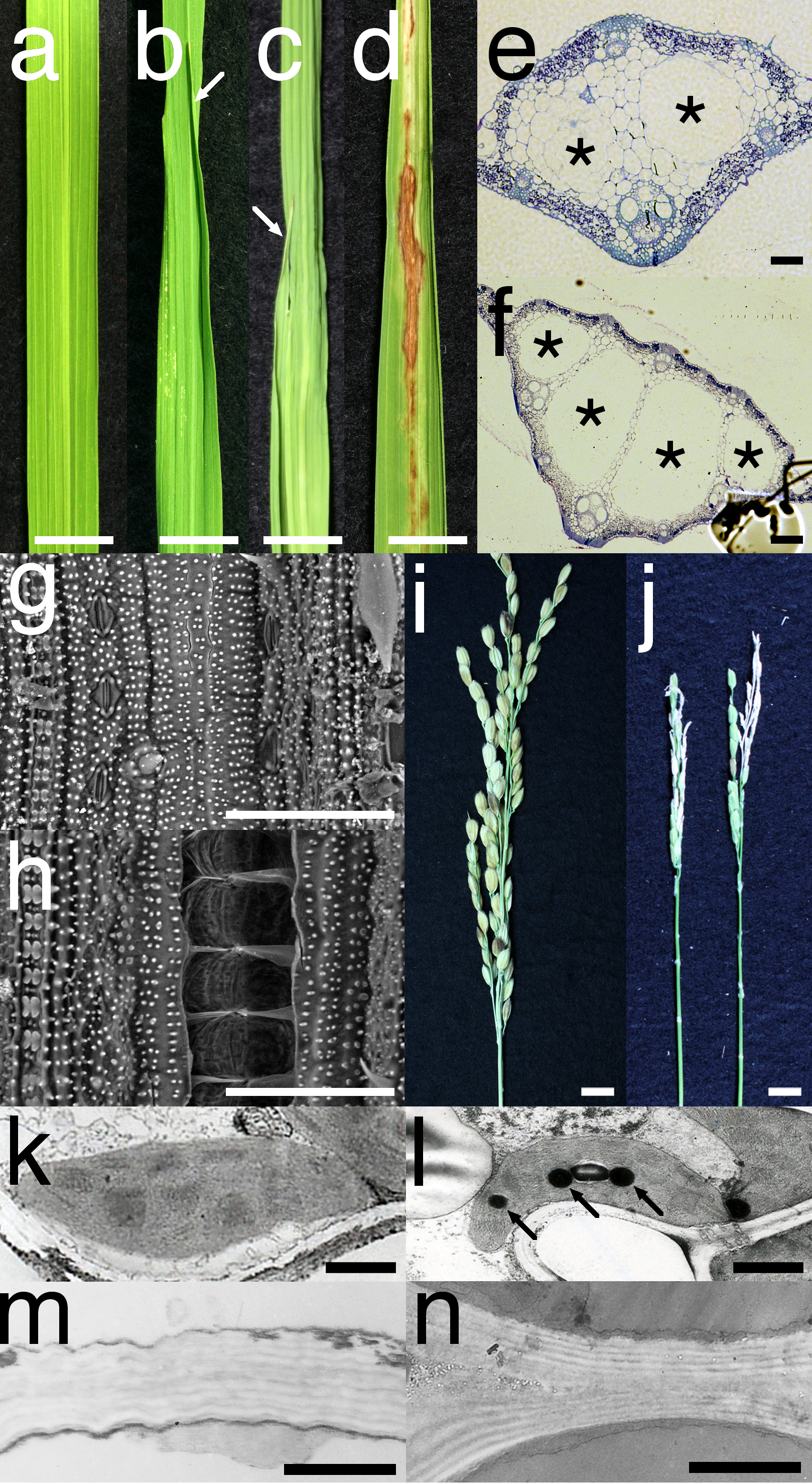

Supplement: Supplementary file 4 — Authors’ original file for figure 3 [file 12284_2012_25_MOESM4_ESM.tiff]

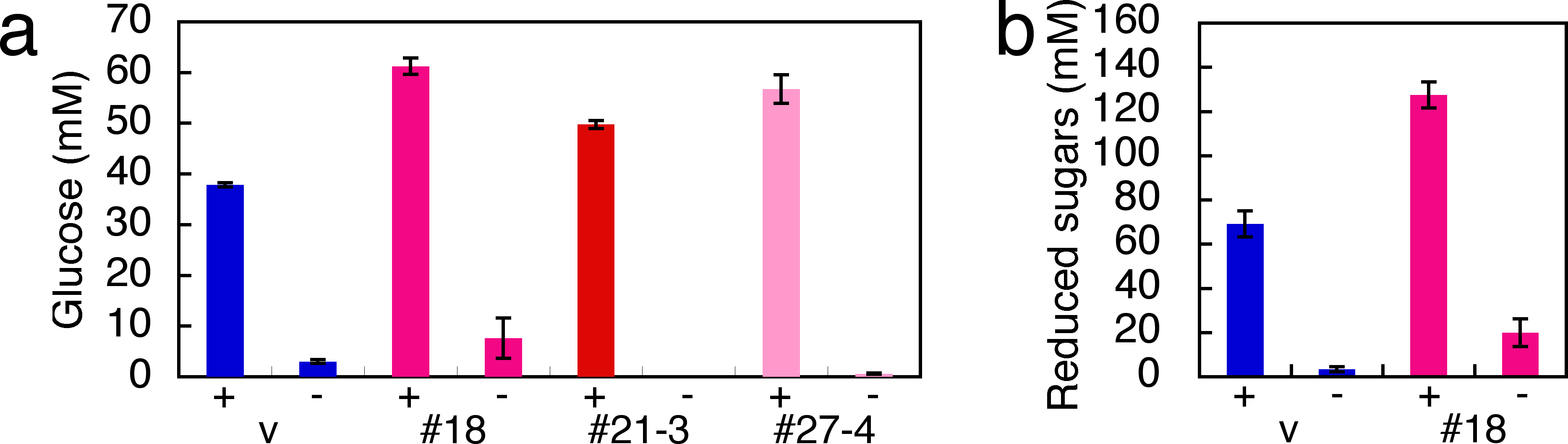

Supplement: Supplementary file 5 — Authors’ original file for figure 4 [file 12284_2012_25_MOESM5_ESM.tiff]
